# Supplementary material for: Cellular Mechanisms Involved in the Combined Toxic Effects of Diesel Exhaust and Metal Oxide Nanoparticles
Source: Nanomaterials (Basel). 2021 May 29;11(6):1437. doi: 10.3390/nano11061437 (PMC8228517; doi:10.3390/nano11061437)
Supplement: Supplementary file 1 [file nanomaterials-11-01437-s001.zip › nanomaterials-1224881-supplementary.pdf]

nanomaterials

MDPI

Supplementary Materials

Cellular Mechanisms Involved in the Combined Toxic Effects of Diesel Exhaust and Metal Oxide Nanoparticles

Alessandra Zerboni [1,*], Rossella Bengalli [1], Luisa Fiandra [1,2], Tiziano Catelani [3] and Paride Mantecca [1]

[1] POLARIS Research Center, Department of Earth and Environmental Sciences, University of Milano—Bicocca, Piazza della Scienza 1, 20126 Milan, Italy; rossella.bengalli@unimib.it (R.B.); luisa.fiandra@unimib.it (L.F.); paride.mantecca@unimib.it (P.M.)

[2] Inter-University Center for the Promotion of the 3Rs Principles in Teaching & Research (Centro 3R), 56122 Pisa, Italy

[3] Microscopy facility, University of Milano-Bicocca, Piazza della Scienza 3, 20126 Milano, Italy; tiziano.catelani@unimib.it

* Correspondence: a.zerboni2@campus.unimib.it; Tel.: +39-02-6448-2916

Table S1. Inductively coupled plasma-optic emission spectroscopy (ICP-OES) analysis of metal dissolution from CuO and ZnO NPs and mixtures

	**ZnO**	**DEP+ZnO**	**CuO**	**DEP+CuO**
3h ppm Zn	9.36 ± 1.1	8.3 ± 0.3	0.1 ± 0.0	0.1 ± 0.0
3h % Zn	51.2 %	51.5 %	0 %	0 %
24 h ppm Zn	11.1 ± 0.1	10.0 ± 0.1	nd	nd
24 h % Zn	69.4 %	62.5 %	0 %	0 %
3h ppm Cu	nd	nd	2.4 ± 0.1	2.4 ± 0.1
3h % Cu	0 %	0 %	14.7 %	14.5 %
24 h ppm Cu	nd	nd	7.1 ± 0.2	4.4 ± 0.2
24 h % Cu	0 %	0 %	43.3 %	26.7 %

The release of ions was evaluated after 3 and 24 h of incubation of NPs (20 μg/mL) and mixtures with DEP (100 μg/mL of DEP and 20 μg/mL of NPs) in cell culture medium. Data were expressed as concentration in ppm ± SE. In the table, percentage of dissolute ions after 3 and 24 h in medium, calculated on the base of ICP-OES analysis, is presented. nd = not detected; Data previously published (Zerboni et al. 2019).

Table S2. Dynamic light scattering (DLS) analyses of CuO and ZnO NPs and mixtures

	**ZnO**	**DEP+ZnO**	**CuO**	**DEP+CuO**
Milli-Q				
Z-average ± ES (nm)	275.7 ± 9	179.7 ± 2	208.43 ± 2	217.07 ± 3
PdI	0.361	0.352	0.209	0.223
Opti-MEM 1% FBS				
Z-average ± ES (nm)	314.38 ± 204	207.37 ± 7	464.67 ± 2	275.7 ± 3
PdI	0.63	0.52	0.35	0.22
Milli-Q				
Z-potential ± ES (mV)	25 ± 0.13	−19 ± 0.21	12 ± 0.6	-18 ± 0.09

Z-average and PdI (polidispersity index) of particles suspended in Milli-Q water and in culture medium, as well as ζ-potential in water, are presented. For the analyses, CuO and ZnO NPs at the concentration of 20 μg/mL were used, while for the mixtures, a suspension of 100 μg/mL of DEP and 20 μg/mL of NPs were prepared. For the analyses of DEP, the concentration of 100 μg/mL was used. Data previously published (Zerboni et al. 2019)

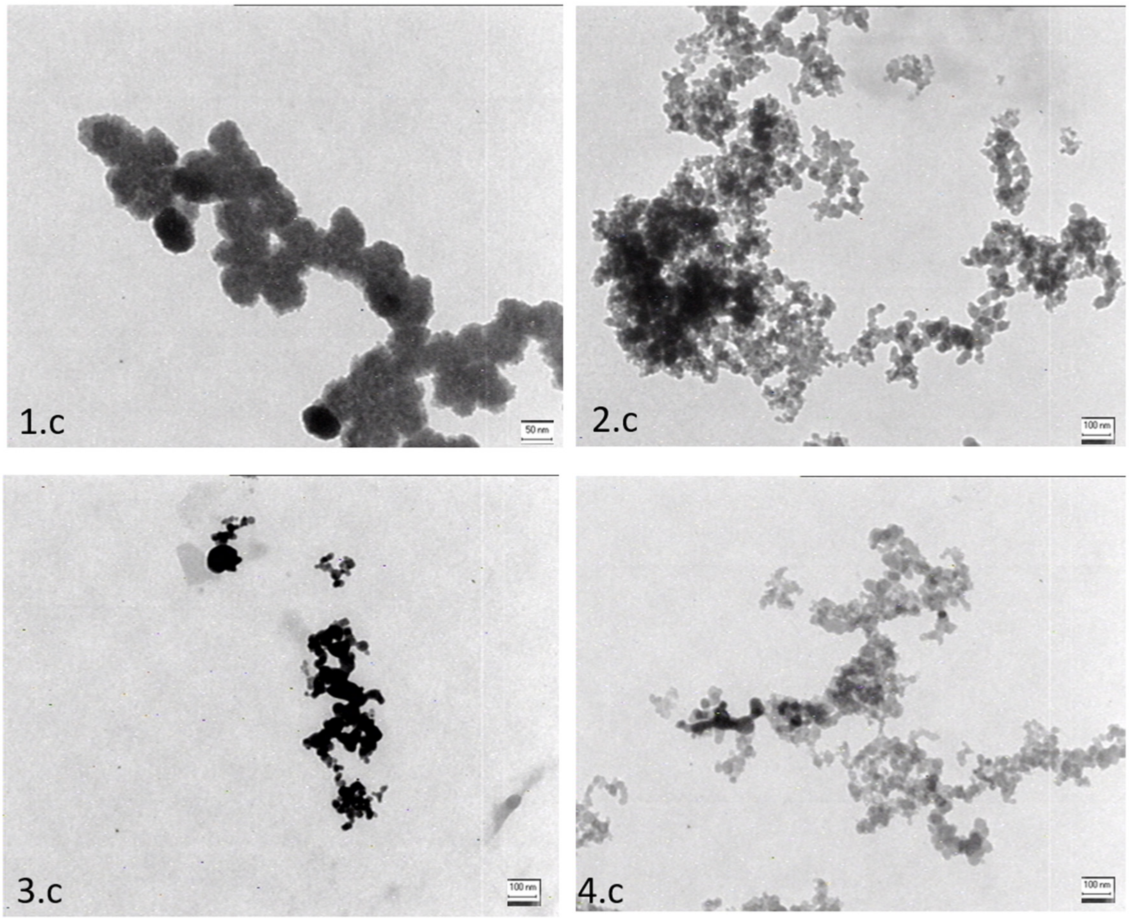

Figure S1. Morphological characterization of particles using Transmission electron microscopy (TEM). ZnO NPs 1.C; DEP + ZnO NPs 2.C; CuO 3.C; DEP + CuO 4.C

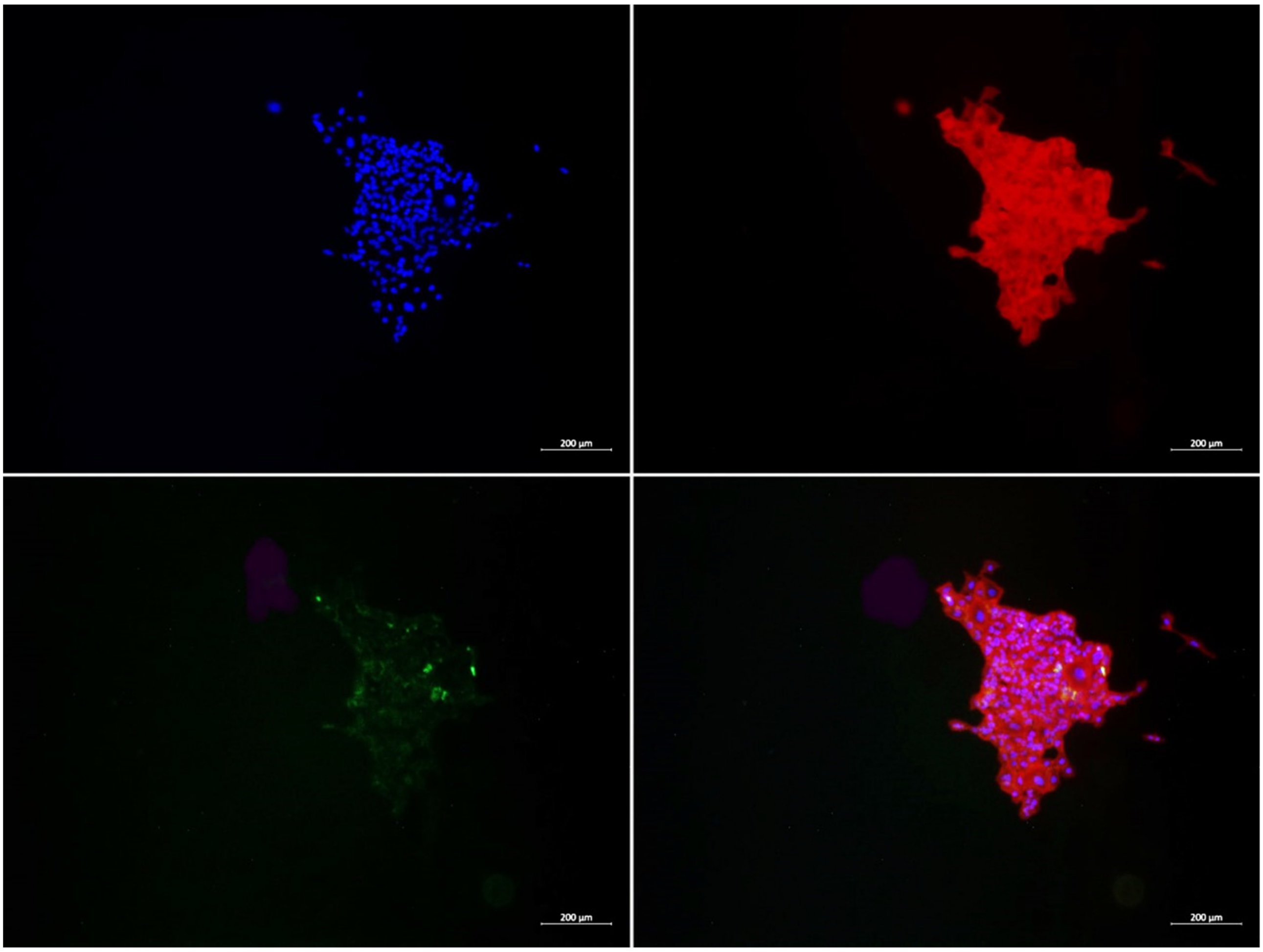

Figure S2. Colony type A. Colonies' classification: E-cadherin (green), phalloidin rhodamine (red) and DAPI (blue) staining of colonies

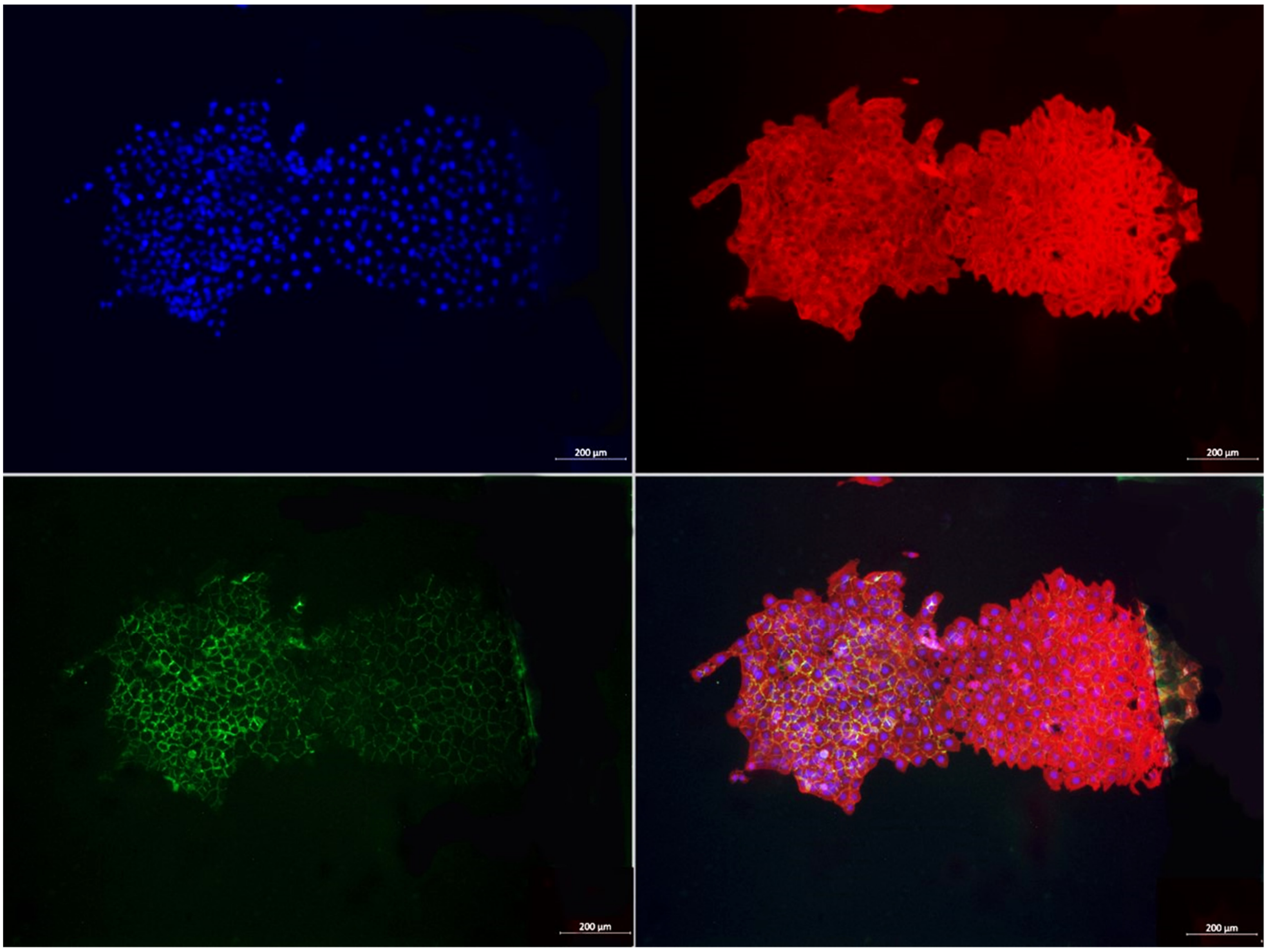

Figure S3. Colony type B. Colonies' classification: E-cadherin (green), phalloidin rhodamine (red) and DAPI (blue) staining of colonies

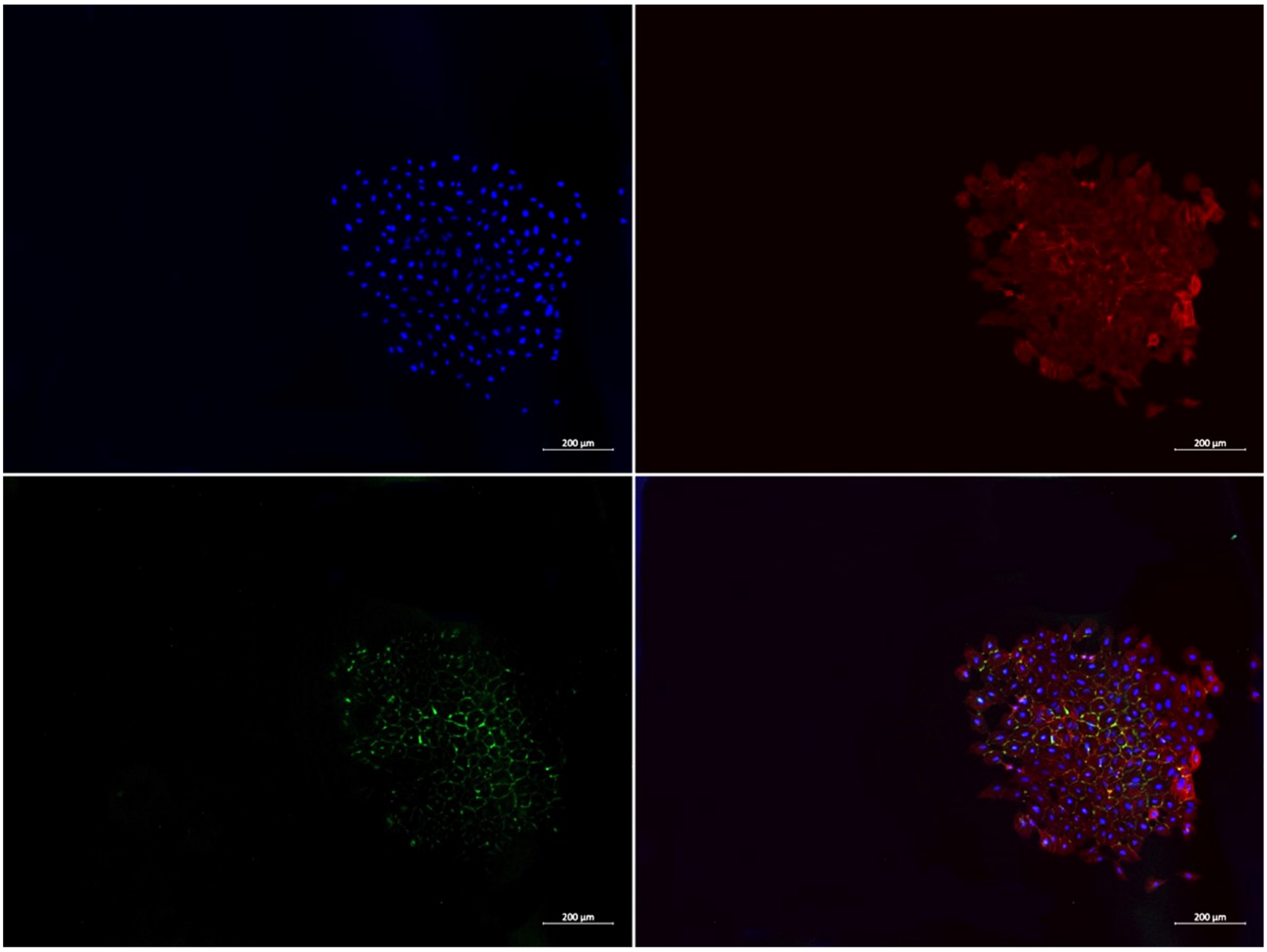

Figure S4. Colony type C. Colonies' classification: E-cadherin (green), phalloidin rhodamine (red) and DAPI (blue) staining of colonies

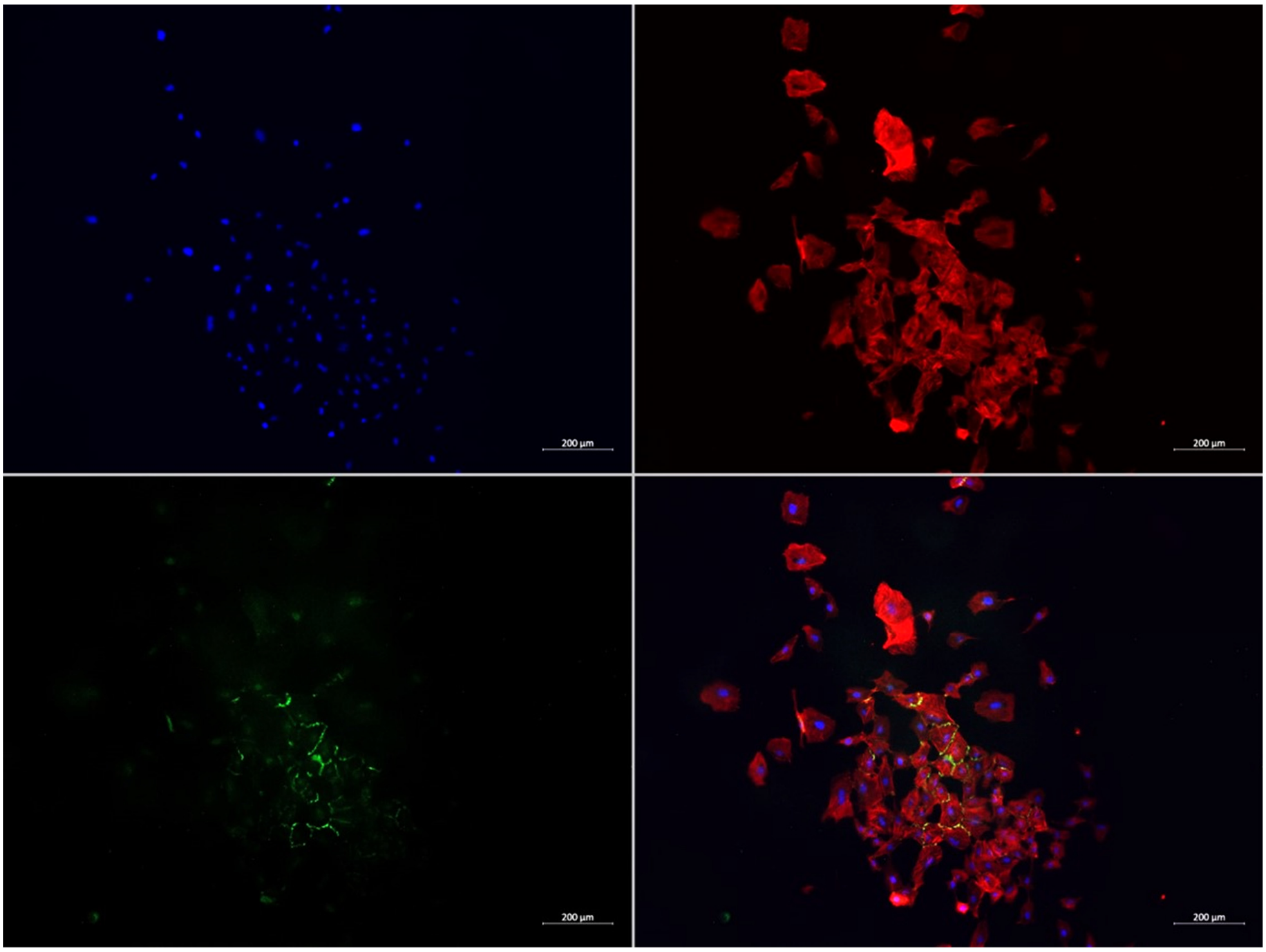

Figure S5. Colony type D. Colonies' classification: E-cadherin (green), phalloidin rhodamine (red) and DAPI (blue) staining of colonies.

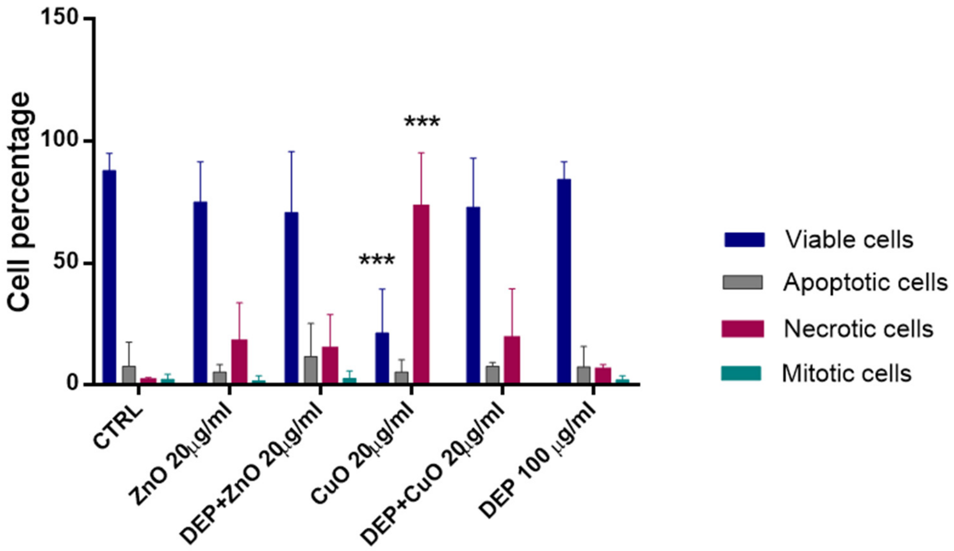

Figure S6. Percentage of viable, apoptotic, necrotic, mitotic cells, according to Hoechst/PI staining, in control cells and cells exposed to ZnO; CuO (20µg/ml); DEP (100 µg/mL) and respective mixtures for 24 h. Statistical analysis was performed by One-way ANOVA with Dunnett's multiple comparisons tests. *** $p < 0.0001$ vs control.

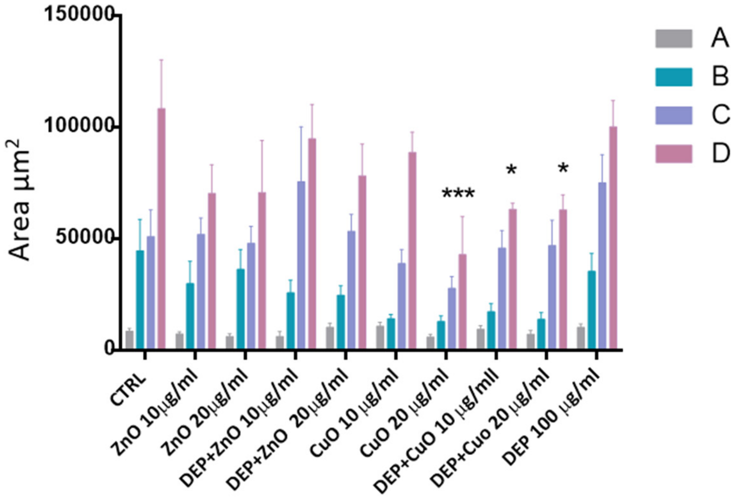

Figure S7. T mean area of colonies (µm²) divided in the four type **(A, B, C, D)** in control cells and cells exposed to ZnO; CuO (20µg/ml); DEP (100 µg/ml) and respective mixtures for 24 h

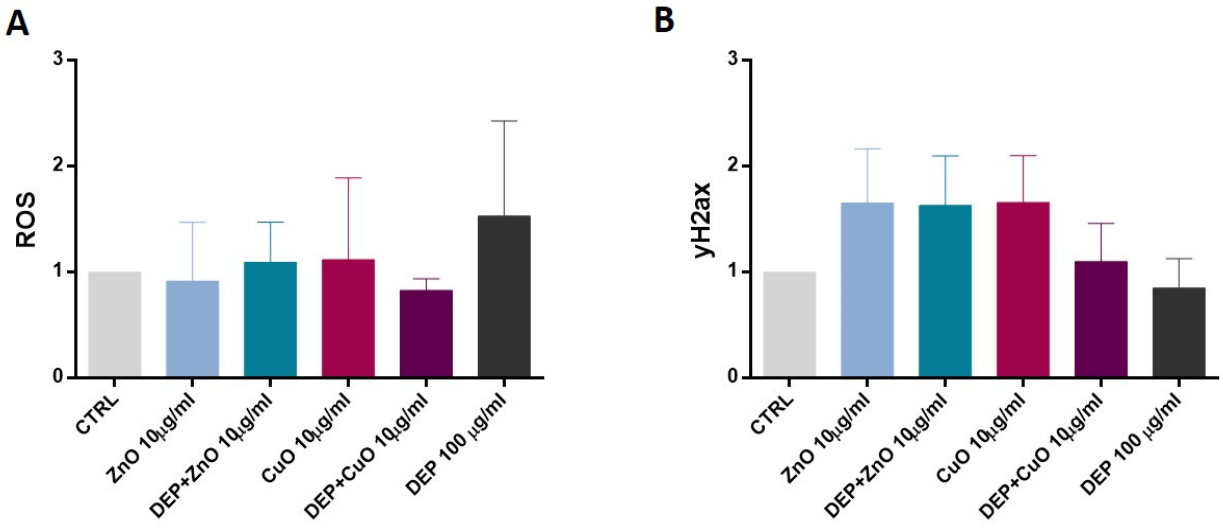

Figure S8. (A) ROS and **(B)** γ-H2AX fold increase in cells exposed to ZnO and CuO NPs (10µg/mL); DEP (100 µg/mL) and DEP+ZnO and DEP+CuO (concentration of NPs: 10 µg/mL) mixtures for 24 h.
